# Protocol digest of a randomized phase III study of pola-R-CHP/high-dose methotrexate/IT vs. pola-R-CHP/IT for newly diagnosed diffuse large B-cell lymphoma with high risk of central nervous system relapse: JCOG2201 (PREMIER)

**DOI:** 10.1093/jjco/hyaf202

**Published:** 2025-12-17

**Authors:** Yusuke Sano, Kana Miyazaki, Motoko Yamaguchi, Senzo Taguchi, Ryunosuke Machida, Kota Kawabata, Haruhiko Fukuda, Kazuyuki Shimada, Hirokazu Nagai, Wataru Munakata, Dai Maruyama, Japan Clinical Oncology Group Lymphoma Study Group

**Affiliations:** Japan Clinical Oncology Group Data Center/Operations Office, National Cancer Center Hospital, 5-1-1 Tsukiji, Chuo-ku, Tokyo 104-0045, Japan; Department of Hematology and Oncology, Mie University Graduate School of Medicine, 2-174 Edobashi, Tsu, Mie 514-8507, Japan; Department of Hematological Malignancies, Mie University Graduate School of Medicine, 2-174 Edobashi, Mie 514-8507, Japan; Department of Radiation Oncology, Cancer Institute Hospital, Japanese Foundation for Cancer Research, 3-8-31 Ariake, Koto-ku, Tokyo 135-8550, Japan; Japan Clinical Oncology Group Data Center/Operations Office, National Cancer Center Hospital, 5-1-1 Tsukiji, Chuo-ku, Tokyo 104-0045, Japan; Japan Clinical Oncology Group Data Center/Operations Office, National Cancer Center Hospital, 5-1-1 Tsukiji, Chuo-ku, Tokyo 104-0045, Japan; Japan Clinical Oncology Group Data Center/Operations Office, National Cancer Center Hospital, 5-1-1 Tsukiji, Chuo-ku, Tokyo 104-0045, Japan; Department of Hematology and Oncology, Nagoya University Graduate School of Medicine, 65 Tsurumai-cho, Showa-ku, Nagoya, Aichi 466-8550, Japan; Department of Hematology, National Hospital Organization Nagoya Medical Center, 4-1-1 Sannomaru, Naka-ku, Nagoya, Aichi 460-0001, Japan; Department of Hematology, National Cancer Center Hospital, 5-1-1 Tsukiji, Chuo-ku, Tokyo 104-0045, Japan; Department of Hematology Oncology, Cancer Institute Hospital, Japanese Foundation for Cancer Research, 3-8-31 Ariake, Koto-ku, Tokyo 135-8550, Japan

**Keywords:** diffuse large B-cell lymphoma, central nervous system, methotrexate, intrathecal injections

## Abstract

Diffuse large B-cell lymphoma (DLBCL) is the most common subtype of non-Hodgkin lymphoma. Central nervous system (CNS) relapse is an uncommon yet lethal event in patients with DLBCL. Polatuzumab vedotin, rituximab, cyclophosphamide, doxorubicin, and prednisolone (pola-R-CHP) is a standard of care for patients with untreated DLBCL. In patients with DLBCL at high risk of CNS relapse, the intrathecal (IT) administration of methotrexate and cytarabine is widely adopted for CNS prophylaxis; however, the optimal treatment is unclear. The aim of this multicenter randomized phase III trial is to confirm the superiority of high-dose methotrexate administered during early cycles of pola-R-CHP in addition to IT chemotherapy for patients with untreated DLBCL at high risk of CNS relapse. A total of 390 patients from 52 institutions will be enrolled across a 4-year period. The primary endpoint is progression-free survival. This trial was registered in the Japan Registry of Clinical Trials as jRCTs031230505 [https://jrct.mhlw.go.jp/latest-detail/jRCTs031230505].

## Teaser

This randomized phase III trial aims to confirm the superiority of high-dose methotrexate administered in addition to pola-R-CHP plus IT chemotherapy for DLBCL with a high risk of CNS relapse.

## Introduction

Diffuse large B-cell lymphoma (DLBCL) is the most common subtype of non-Hodgkin lymphoma, accounting for ~30% of all malignant lymphomas [1, 2]. Central nervous system (CNS) relapse occurs in ~5% of patients with DLBCL [3–6], and this event is associated with poor prognosis [7].

The addition of rituximab, an anti-CD20 monoclonal antibody, to cyclophosphamide, doxorubicin, vincristine, and prednisone (R-CHOP) has been established as the standard first-line treatment for DLBCL since the early 2000s. In 2021, the POLARIX study demonstrated the superiority of polatuzumab vedotin, an anti-CD79b monoclonal antibody conjugated by a protease-cleavable linker to monomethyl auristatin E, plus rituximab, cyclophosphamide, doxorubicin, and prednisone (pola-R-CHP) over R-CHOP in terms of progression-free survival (PFS) (2-year PFS, 76.7% vs. 70.2%; stratified hazard ratio, 0.73 [95% confidence interval (CI), 0.57–0.95; *P* = .02) [8]. Therefore, pola-R-CHP is a standard first-line treatment for untreated DLBCL. In the POLARIX study, CNS relapse was reported in 3.0% of patients in the pola-R-CHP arm and in 2.7% of those in the R-CHOP arm, indicating comparable incidences of CNS relapse between the two regimens [8].

Despite these therapeutic advances, CNS relapse remains an unresolved clinical challenge in the treatment of DLBCL. While the overall incidence of CNS relapse is ~5%, the risk in certain high-risk subgroups exceeds 10%. Risk factors for CNS relapse include a CNS International Prognostic Index (CNS-IPI) score indicating high risk [3]; the involvement of one or more specific high-risk extranodal sites (testis, uterus, ovary, fallopian tube, breast, orbit, nasal cavity, paranasal sinus, kidney, adrenal gland, bone marrow, or bone) [5, 9–13]; and the presence of multiple extranodal sites of involvement [14].

CNS prophylaxis with intrathecal (IT) administration of methotrexate and cytarabine is the standard treatment for acute lymphoblastic leukemia and adult T-cell leukemia/lymphoma. Furthermore, for primary testicular DLBCL, the Japanese Hematopoietic Tumor Treatment Guidelines [15] recommend the administration of R-CHOP with CNS prophylaxis by IT chemotherapy, followed by prophylactic radiotherapy to the contralateral testis on the basis of the results of a phase II study [16]. IT prophylaxis is widely adopted as a community standard in clinical practice. However, the current treatment efficacy remains unsatisfactory.

Given that high-dose methotrexate (HD-MTX) is commonly used in the treatment of primary CNS lymphoma, it is anticipated that HD-MTX will reduce the risk of CNS relapse in patients with DLBCL. Retrospective studies evaluating the use of HD-MTX for CNS prophylaxis have reported conflicting results, possibly due to the presence or absence of IT chemotherapy. Studies have shown that the combination of HD-MTX with IT chemotherapy reduces the incidence of CNS relapse [17, 18]; however, HD-MTX alone has not been shown to reduce the incidence of CNS relapse [19–21]. These findings indicate that the combination of HD-MTX and IT chemotherapy is necessary to achieve effective CNS prophylaxis.

Furthermore, the optimal timing of HD-MTX administration also requires careful consideration. The median time from initial DLBCL diagnosis to CNS relapse is reported to be 8–8.5 months, indicating that CNS relapse often occurs during or shortly after first-line chemotherapy [3, 4, 22]. Thus, early initiation of CNS-directed prophylaxis is critical. PRIMUEUR-IVL demonstrated promising clinical outcomes in patients with untreated intravascular large B-cell lymphoma without apparent CNS involvement who were treated with HD-MTX during the first and last three cycles of R-CHOP in addition to IT chemotherapy (2-year PFS, 76% [95% CI, 58–87]; 2-year cumulative incidence of secondary CNS recurrence, 3% [95% CI, 0.2–12]) [23].

Hence, we designed a multicenter randomized controlled phase III trial to confirm whether HD-MTX administered between the first and last three cycles of pola-R-CHP in addition to IT chemotherapy can prolong PFS in patients with DLBCL who are at high risk of CNS relapse. The study protocol was approved by the Certified Review Board of the National Cancer Center Hospital in October 2023. This trial was registered in the Japan Registry of Clinical Trials with trial number jRCTs031230505 [https://jrct.mhlw.go.jp/latest-detail/jRCTs031230505].

## Protocol digest of JCOG2201

### Objectives

This trial aims to confirm the superiority of pola-R-CHP/HD-MTX/IT chemotherapy (Arm B) over pola-R-CHP/IT chemotherapy (Arm A) in terms of PFS and that pola-R-CHP/HD-MTX/IT chemotherapy is not inferior in terms of overall survival (OS) in patients with DLBCL who are at high risk of CNS relapse.

### Study setting

**Figure 1:**
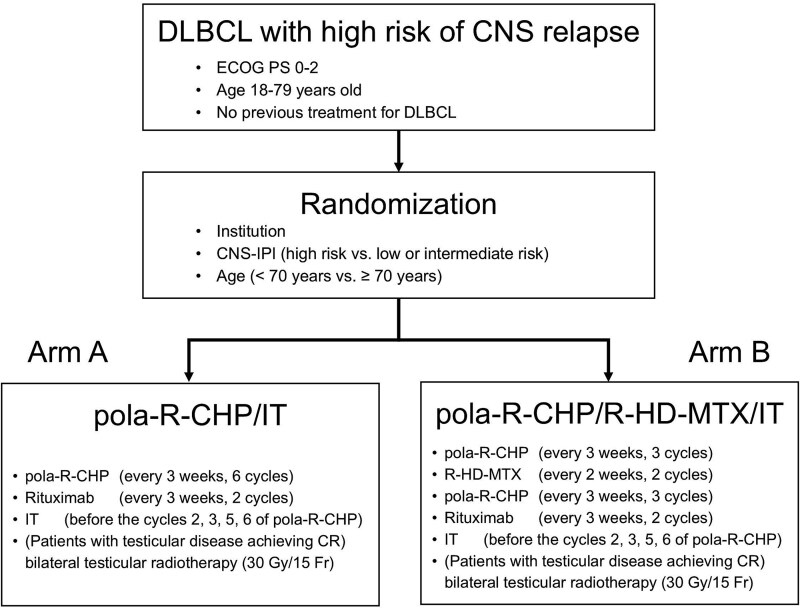
Study schema of JCOG2201.

This is a multi-institutional, randomized, phase III trial. A schematic of the trial is illustrated in Fig. 1.

### Endpoints

The primary endpoint is PFS, which is defined as the time from the date of registration to the date of disease progression or death from any cause, and data will be censored at the last day when a patient is alive without any evidence of progression. The secondary endpoints are OS, cumulative incidence of CNS relapse, response rate, complete response rate, and incidence of adverse events. OS is defined as the time from the date of registration to the date of death from any cause, and data will be censored on the last day when a patient is alive. Toxicity will be evaluated according to the Common Terminology Criteria for Adverse Events (CTCAE) v5.0.

### Inclusion criteria

Patients who meet all of the following criteria are enrolled in this trial:

1) Pathologically proven mature B-cell neoplasms, except neoplasms with histological transformation from lymphoid tumors other than DLBCL, according to the World Health Organization (WHO) classification (2017).

(i) DLBCL, not otherwise specified (NOS)
(ii) T-cell/histiocyte-rich large B-cell lymphoma
(iii) Primary cutaneous DLBCL, leg type
(iv) EBV-positive DLBCL, NOS
(v) ALK-positive large B-cell lymphoma

2) No diagnosis of high-grade B-cell lymphoma on the basis of FISH or chromosome testing. If treatment needs to be started immediately, a patient may be registered before the test results are confirmed.
3) CD20 positivity.
4) Meeting any of the following ((i) to (iii)):

(i) CNS-IPI score of 4–6 (high-risk group; Table 1).
(ii) CNS-IPI score of 0–3 (low/intermediate-risk group; Table 1), with the involvement of one or more organs at high risk of CNS relapse (testis, uterus, ovary, fallopian tube, breast, orbit, nasal cavity, paranasal sinus, kidney, adrenal gland, bone marrow, or bone).
(iii) CNS-IPI score of 0–3 (low/intermediate-risk group), with no lesions of the organs at high risk of CNS relapse and 3 or more extranodal lesions.

5) Age of 18–79 years.
6) Eastern Cooperative Oncology Group (ECOG) performance status (PS) of 0–2.
7) The presence or absence of measurable lesions is not required
8) No CNS involvement.
9) No previous chemotherapy, radiotherapy, interferon-alpha therapy, or antibody therapy for DLBCL.
10) No previous immunosuppressant treatment.
11) Sufficient organ function assessed within 14 days prior to registration

(i) Peripheral blood tumor cell count ≥1.0 × 10^4^/mm^3^
(ii) Absolute neutrophil count ≥1000/mm^3^
(iii) Platelet count ≥7.5 × 10^4^/mm^3^ (≥5.0 × 10^4^/mm^3^ in patients with lymphoma invasion of the bone marrow)
(iv) Total bilirubin level ≤2.0 mg/dl
(v) Aspartate aminotransferase (AST) level ≤ 100 U/L
(vi) Alanine aminotransferase (ALT) level ≤ 100 U/L
(vii) Serum creatinine level ≤1.5 mg/dL
(viii) SpO_2_ ≥93% (room air)

12) No ischemic change, atrial fibrillation, or ventricular arrhythmia requiring treatment shown on the latest ECG (assessed within 28 days prior to registration).
13) Left ventricular ejection fraction ≥50% (assessed within 56 days prior to registration).
14) Written informed consent.

**Table 1:** Central nervous system international prognostic index (CNS-IPI).

**Factor**	**Criteria (1 point each)**
Age	>60 years
Serum LDH	>Upper limit of normal
ECOG PS	>1
Ann Arbor stage	III or IV
Number of extranodal sites	>1
Kidney and/or adrenal gland involvement	Present
**Risk group**	**Score**
Low	0–1
Intermediate	2–3
High	4–6

LDH, lactate dehydrogenase; ECOG PS, Eastern Cooperative Oncology Group performance status.

### Exclusion criteria

Patients who meet any of the following criteria are excluded from this trial:

1) Synchronous double or multiple cancers or metachronous double or multiple cancers with a progression-free period of <5 years.
2) Infectious disease requiring systemic treatment.
3) A fever greater than 38° (except when infection or drug fever can be ruled out).
4) Pregnancy, 28 days postparturition or lactation (females) and partner pregnancy (males).
5) Severe psychological disorders.
6) The receipt of continuous systemic corticosteroid or immunosuppressant treatment.
7) Uncontrolled diabetes mellitus.
8) Uncontrolled hypertension.
9) Unstable angina pectoris or a history of myocardial infarction within 6 months.
10) Uncontrolled valvular heart disease, dilated cardiomyopathy, or hypertrophic cardiomyopathy.
11) HBs antigen or HCV antibody positivity.
12) HIV antibody positivity.
13) Interstitial pneumonia, pulmonary fibrosis, or severe pulmonary emphysema shown on chest X-ray.

### Randomization

After the eligibility criteria are confirmed, registration is performed using a web-based system at the JCOG Data Center. The patients are randomized (1:1) to Arm A or Arm B. The minimization method with a random component will be used for randomization according to institution, CNS-IPI (high risk vs. low or intermediate risk), and age (<70 years vs. ≥70 years).

### Treatment methods

#### Arm A: Pola-R-CHP/IT chemotherapy

Patients who are allocated to Arm A receive six cycles of pola-R-CHP (day 1, intravenously: polatuzumab vedotin, 1.8 mg/kg; rituximab, 375 mg/m^2^; cyclophosphamide, 750 mg/m^2^; and doxorubicin, 50 mg/m^2^; and days 1–5, orally: prednisolone, 100 mg/body [40 mg/m^2^ for those aged 65 years or older at the time of registration]) every three weeks, followed by two cycles of rituximab (375 mg/m^2^ intravenously on day 1) every three weeks. IT chemotherapy with methotrexate (15 mg), cytarabine (40 mg), and prednisolone (10 mg) is administered before the start of pola-R-CHP cycles two, three, five, and six. Patients with testicular disease who achieve complete response after chemotherapy receive bilateral testicular radiotherapy (30 Gy/15 Fr). Granulocyte colony-stimulating factor (either pegfilgrastim, filgrastim, or lenograstim) is administered as primary prophylaxis in each pola-R-CHP cycle.

#### Arm B: Pola-R-CHP/R-high-dose methotrexate/IT chemotherapy

Patients who are allocated to Arm B receive three cycles of pola-R-CHP followed by two cycles of R-HD-MTX (rituximab, 375 mg/m^2^ intravenously on day 1 and methotrexate, 3.5 g/m^2^ [2.0 g/m^2^ for those aged 70 years or older at the time of registration] intravenously on day 2) every two weeks and three additional cycles of pola-R-CHP. IT chemotherapy with methotrexate (15 mg), cytarabine (40 mg), and prednisolone (10 mg) is administered before the start of pola-R-CHP cycles two, three, five, and six. Patients with testicular disease who achieve complete response after chemotherapy receive bilateral testicular radiotherapy (30 Gy/15 Fr). To reduce HD-MTX toxicity, patients receive leucovorin (24 mg) intravenously a total of 12 times (every 6 h beginning 24 h after HD-MTX initiation). If the concentration of methotrexate in the blood is 1 μmol/L or higher 48 h after HD-MTX initiation or 0.1 μmol/L or higher at 72 h after initiation, we ensure adequate hydration, alkalinization of the urine, increased administration of leucovorin, and prolonged leucovorin rescue until the concentration is <1 μmol/L at 48 h and 0.1 μmol/L at 72 h, respectively.

### Follow-up

All patients enrolled in this trial will be followed for at least 5 years after the completion of patient accrual, with the primary analysis conducted 2 years after accrual completion. Physical and blood examinations will be performed every 2 or 3 months for the first 2 years, every 6 months for the subsequent 3 years, and annually after the 5-year follow-up. Echocardiography will be performed within 6 months and at 2 and 5 years after the end of chemotherapy. Contrast-enhanced computed tomography (CT) of the head, cervix, chest, abdomen, and pelvis, as well as soluble IL-2 receptor assessment, will be performed every 6 months for the first 5 years and annually after the 5-year follow-up. Fludeoxyglucose positron emission tomography/CT will be conducted at the end of the protocol treatment. Histopathology biopsy, bone marrow biopsy, gastrointestinal endoscopy, or bone X-ray/magnetic resonance imaging will be conducted as necessary.

### Study design and statistical analysis

This trial was designed to confirm the superiority of pola-R-CHP/high-dose methotrexate/IT chemotherapy (Arm B) over pola-R-CHP/IT chemotherapy (Arm A) in patients with DLBCL who are at high risk of CNS relapse. We assume that the 2-year PFS in Arm A will be 70% according to the results of JCOG0601 [24] and the POLARIX study [8] and expect an 8% increase in Arm B (hazard ratio of 0.70). According to Schoenfeld and Richter’s method [25], the required sample size (number of PFS events) was calculated as 380 (167) in total, with a one-sided alpha level of 5%, a power of 75%, an expected accrual period of 4 years, and a follow-up period of 2 years. The planned sample size was set at 390 patients to account for lost to follow-up. The primary analysis for PFS will be performed based on an intention-to-treat basis, and the stratified log-rank test with the CNS-IPI score and age as the stratification factors will be used. If Arm B is superior to Arm A in terms of PFS and not inferior in terms of OS, we will conclude that the treatment used in Arm B is better. The stratified Cox proportional hazard model is used to estimate the hazard ratio between the arms. All the statistical analyses will be conducted at the JCOG Data Center.

### Interim analysis and monitoring

Two interim analyses are planned. The first will be conducted after enrolling half of the planned number of patients, and the second will be conducted after achieving the planned patient accrual and completing the patients’ protocol treatment. The Lan-DeMets alpha spending function with the O’Brien and Fleming type will be used to adjust the multiplicity [26]. Study termination due to efficacy will be considered if PFS of Arm B is superior to that of Arm A according to the adjusted significance level of alpha. The conditional power [27] and predictive probability [28] for the primary endpoint will be used to judge whether the study should be terminated because of futility. The JCOG Data and Safety Monitoring Committee will review the interim analysis report independently from the group investigators and group statistician to determine whether the study should be terminated early.

The JCOG Data Center, Study Coordinator, and Site Investigators responsible for the day-to-day management of the study will conduct central monitoring and issue a report every 6 months to evaluate the progress of the study and improve data integrity and patient safety. For quality assurance, site visit audits will be performed by the JCOG Audit Committee (not on a study-specific basis but for the study group).

## Participating institutions (from north to south)

Sapporo Hokuyu Hospital, Iwate Medical University, Tohoku University Hospital, Akita University Graduate School of Medicine, Yamagata University Hospital, Faculty of Medicine, University of Tsukuba, Gunma University, Saitama Medical University International Medical Center, Saitama Medical Center, Saitama Medical University, National Cancer Center Hospital East, Chiba Cancer Center, National Cancer Center Hospital, Kyorin University Faculty of Medicine, Tokyo Metropolitan Cancer and Infectious diseases Center Komagome Hospital, Keio University Hospital, Jikei University Hospital, Jikei University, Daisan Hospital, Cancer Institute Hospital of Japanese Foundation for Cancer Research, Tokai University School of Medicine, Kanagawa Cancer Center, University of Fukui Hospital, Gifu University School of Medicine, Hamamatsu University School of Medicine, Aichi Cancer Center Hospital, National Hospital Organization Nagoya Medical Center, Nagoya University School of Medicine, Fujita Health University, Nagoya City University Hospital, Japanese Red Cross Aichi Medical Center Nagoya Daini Hospital, Aichi Medical University, Toyota Kosei Hospital, Mie University School of Medicine, University Hospital Kyoto Prefectural University of Medicine, Japanese Red Cross Kyoto Daiichi Hospital, Osaka University Graduate School of Medicine, Kindai University Hospital, Osaka International Cancer Institute, Hyogo Medical University, Hyogo Cancer Center, Wakayama Medical University, School of Medicine, Shimane University Faculty of Medicine, National Hospital Organization Shikoku Cancer Center, National Kyushu Cancer Center, School of Medicine, Fukuoka University, University of Occupational and Environmental Health, Nagasaki Medical Center, Sasebo City General Hospital, Nagasaki University Hospital, Kumamoto University Hospital, NHO Kumamoto Medical Center, Oita Prefectural Hospital, Kagoshima University Hospital.
